# Fingerprint Sweat Pore Density in Patients with Oligodontia: A Controlled Clinical Trial

**DOI:** 10.3390/biomedicines12122768

**Published:** 2024-12-05

**Authors:** Jonas Q. Schmid, Jens Reimann, Claudius Middelberg, Ole Oelerich, Thomas Stamm, Ariane Hohoff

**Affiliations:** 1 Department of Orthodontics, University of Münster, 48149 Münster, Germany; 2 Private Practice, 48336 Sassenberg, Germany; 3 Department of Cranio-Maxillofacial Surgery, University of Münster, 48149 Münster, Germany

**Keywords:** tooth agenesis, sweat pore, oligodontia, hypodontia, ectodermal dysplasia

## Abstract

**Background/Objectives:** There is a lack of evidence for the relationship between sweat pores and tooth agenesis. The aim of this study was to compare sweat pore density on fingertips between a group of patients with oligodontia and a control group without tooth agenesis. **Methods:** This parallel-group controlled clinical trial included 28 patients. Fourteen patients (f/m 9/5; mean age 13.5 ± 3.5 years) with ≥6 congenitally missing permanent teeth, excluding third molars (M3), were enrolled in the study group. The matched control group consisted of 14 patients (f/m 9/5; mean age 12.8 ± 1.8 years) without tooth agenesis. Impressions of 168 fingertips (left and right index, middle, and ring fingers) of the participating subjects were taken and examined using a scanning electron microscope with a 5.85 mm × 4.29 mm region of interest at the center of the fingertip. The primary outcome was the pore-to-pore distance (μm) on a dermal ridge, and the secondary outcome was the number of sweat pores per cm^2^, while pore numbers were adjusted for individual body surface area (BSA). **Results:** There were no statistically significant differences in age, height, weight, and BSA between the groups. The study group had 11.07 ± 4.03 missing teeth, excluding M3. There was a statistically significant difference (*p* = 0.006) in the distance between adjacent pores on a dermal ridge between the study and control groups (354.89 ±32.41 μm vs. 340.31 ±39.04 μm). The unadjusted pore numbers showed a statistically significant difference between the groups, but after adjustment for BSA, this difference was no longer present. **Conclusions:** Patients with oligodontia differed from subjects without tooth agenesis in the distance between two adjacent sweat pores on a dermal ridge. However, the differences were small and of limited clinical significance. Increased pore distance appears to be a better predictor of oligodontia/ectodermal dysplasia than pore number.

## 1. Introduction

Sweat pores are openings of the sweat glands and can be seen on the papillary lines of the fingertips with appropriate magnification. They develop from the basal layer of the epidermis between the 10th and 14th week of gestation and are involved in the formation of the primary ridges through their proliferation pressure [1]. The pattern of primary ridges determines the final pattern of the epidermal ridges and, thus, the distribution of sweat glands. From the 19th week of gestation, the development of the primary ridges is complete and although not yet fully visible, the geometry of the ridge system is already set for life [1]. By the 24th week of gestation at the latest, the sweat glands have also developed [2] and are consequently fixed in number. In addition to primary ridges, secondary ridges also form, but these do not carry sweat spores. The appearance of epidermal ridges may change over a lifetime, but the geometry remains the same [1]; thus, ridges and sweat pores have great discriminatory power.

Sweat pores can be examined in terms of their number, shape, size, position, type, and consecutive distance from each other [2]. These third-level details can be used not only for personal identification [3] but also for diagnostic purposes like psychophysiological measures of autonomic nervous system activity [4], hypohydrosis or anhydrosis [5], tooth size [6], hypodontia [7], ectodermal dysplasia [8], and carrier screening [9].

The fingertips allow pore-specific quantitative analysis in a well-defined area of the skin. The method required is quick and easy to perform, completely safe for the patient, and provides a consistently high-quality sweat pore visualization sufficient for evaluation. On the fingertips, papillary lines and their corresponding grooves form a characteristic pattern of loops and swirls that is unique to each individual. Sweat pores are approximately 66–287 μm in size on the papillary lines of the fingertips [10] and are formed by the excretory ducts of serous glands in the skin. They form small, characteristic indentations on the papillary lines of the fingertips and can, therefore, be well recognized and quantified.

Therefore, the aim of the present study was to compare the sweat pore density on the fingertips between a group of patients with oligodontia and a healthy matched control group without tooth agenesis. The null hypothesis was tested that there is no difference in the pore-to-pore distance on dermal ridges between patients with oligodontia and the control group without tooth agenesis.

## 2. Materials and Methods

This prospective controlled trial was approved by the local Ethics Commission of the Medical Faculty of the University of Münster, Germany (0IIIEhm/). The study protocol was described according to the STROBE Guidelines [11]. The study, including patient recruitment, was conducted at the Department of Orthodontics, University Hospital Münster, Germany. Two groups were formed to assess possible relations between pore density and the presence of missing teeth: a group of patients with oligodontia and a matched control group of patients without dental agenesis.

A sample size calculation using G*Power 3.1 [12] based on α=0.05 (two-sided) and power of 1−β=0.80 was performed. Assuming a clinically meaningful group difference in the distance between pores of 50 μm (SD 40 μm [13]), an effect size (Cohen’s d) of 1.25 was calculated. These values suggested that each group required a minimum of 12 participants. Considering a potential dropout rate of 10%, the sample size was adjusted to 14 participants per group.

Study participants were recruited from consecutively treated patients at the Department of Orthodontics. Inclusion criteria were patients with ≥6 congenitally missing permanent teeth, excluding third molars (M3) [14]. Tooth agenesis was diagnosed on panoramic radiographs. For the matched control group, orthodontically treated patients with the presence of all permanent teeth except M3 were included. Exclusion criteria were patients with calluses, cuts, or scars on the fingertips. Further exclusion criteria were genodermatoses and other self-reported skin disorders that could affect the fingertip ridges. Patients with hypodontia due to extractions, clefts, or craniofacial syndromes with and without associated systemic disorders were also excluded. All patients and their parents or guardians gave informed consent to participate in the study.

Data collection included a clinical examination, recording of age and sex using a questionnaire, and determination of height, weight and the number of missing teeth. The index (r2, l2), middle (r3, l3), and ring fingers (r4, l4) of the right and left hands of each participant were used to count sweat pores. For this purpose, the subject’s hand was first washed twice and then degreased with pure alcohol. An impression of each fingertip was then taken with a low-viscosity dental silicone (Provil novo Light, Kulzer, Hanau, Germany). After a setting time of 4 minutes, the silicone was removed from the fingers. The impressions were then punched out using a circular punch tube with a diameter of 7 mm. The specimens were stored in individually labeled containers until further processing in the laboratory. The addition-curing silicone impression material ensured that there was no dimensional change in the specimens during the storage period.

**Figure 1:**
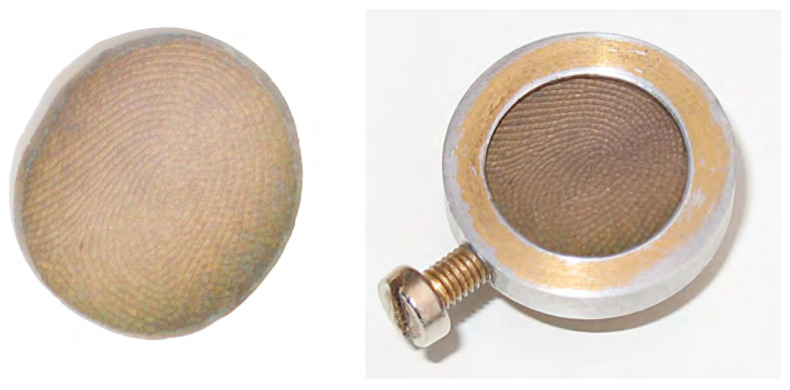
(**Left**): Circular gold sputtered silicone impression of a fingertip. (**Right**): Object holder with circular opening for the specimen.

Surface images of the specimens were then acquired using a scanning electron microscope (PSEM 500, Philips, Amsterdam, The Netherlands). In order to examine the specimens in an identical manner, a special electrically conductive object holder was made to bring the curved impressions into a plane and securely fix them (Figure 1). To obtain an electrically conductive surface of the specimens, the silicone impressions were sputtered with gold (SCD 050, BAL-TEC Inc., Balzers, Liechtenstein).

**Figure 2:**
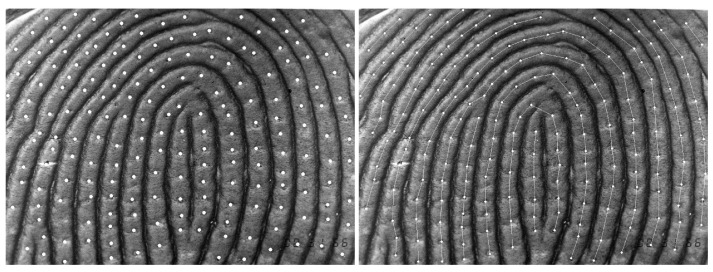
(**Left**): Marking of the pores using the multi-point tool of ImageJ in preparation for automatic counting. 189 pores were counted in an ROI of 25.09 mm^2^, corresponding to a pore density of 753.29 per cm^2^. (**Right**): The distance between consecutive pores was measured with the straight-line tool in ImageJ. In total, 177 lines were drawn and a mean distance of 60.62 pixels was measured, corresponding to a mean distance of 350 μm between consecutive sweat pores on papillary lines.

When generating the images, it was ensured that the region of interest (ROI) always had a width of 5.85 mm and a height of 4.29 mm, corresponding to a skin surface of 25.09 mm^2^. The center of the fingertip was selected as the center of the ROI in each case. The images were loaded into ImageJ 1.53, an image analysis tool [15], for further processing. Window width and window level were adjusted for better recognition of the individual pores. Each image had a resolution of 1050×770 pixels. Knowing the width and height of the ROI, the scale was 179.54 pixels/mm. To automate the series of commands, a simple program was written with the macro language of ImageJ. Using these macros, the individual pores per image were marked and counted (Figure 2, left). The mean distance between consecutive pores of each fingerprint was calculated by connecting adjacent pores using ImageJ’s measuring straight line tool (Figure 2, right).

After evaluation of the images, the number of pores per cm^2^ was adjusted by the body surface area (BSA). This was necessary because the subjects were in different growth stages at the time of the examination. Since no new sweat pores are formed on the skin after birth, the pores drift apart as BSA increases, and the more the individual grows, the more the pores drift apart. From all available estimations of BSA published in the scientific literature [16], only the study by Haycock and coworkers [17] included children. Therefore, Haycock’s equation was used to adjust the number of sweat pores with the following formula: *BSA* (m^2^) = *weight* (kg)^0.5378^ × *height* (cm)^0.3964^ × 0.024265.

### Statistical Analysis

Statistical analysis was performed using SPSS Statistics 29 (IBM Corp., Armonk, NY, USA). Descriptive statistics were calculated for all variables, and Mann–Whitney U tests were used to evaluate differences in the baseline characteristics. To establish a control group matched for age and gender, a case–control matching procedure with a tolerance of 6.5 years in SPSS was used. To examine the potential variation in pore density between the groups, the following calculations were performed: (a) determination of the number of pores per finger, (b) measurement of the number of pores per square centimeter adjusted by body surface area (BSA), (c) measurement of the number of lines between two consecutive pores, and (d) assessment of the distance (μm) between two consecutive pores on a dermal ridge. One-way analysis of variance (ANOVA) was performed to assess the variation in pore density between the fingers of each participant, and differences in pore density between the groups were further analyzed using nonparametric Mann–Whitney U tests. Intrarater reliability for the distance measurements was evaluated using intraclass correlation coefficients (ICC). For this purpose, 10% of the sample was randomly selected using a random number generator and re-evaluated by the principal investigator after at least 4 weeks. ICC estimates and their 95% confidence intervals were calculated based on a single measurement, absolute-agreement, two-way mixed effects model. Interpretation of the correlation coefficients followed the cut-off limits of Koo and Li 2016 [18].

## 3. Results

**Figure 3:**
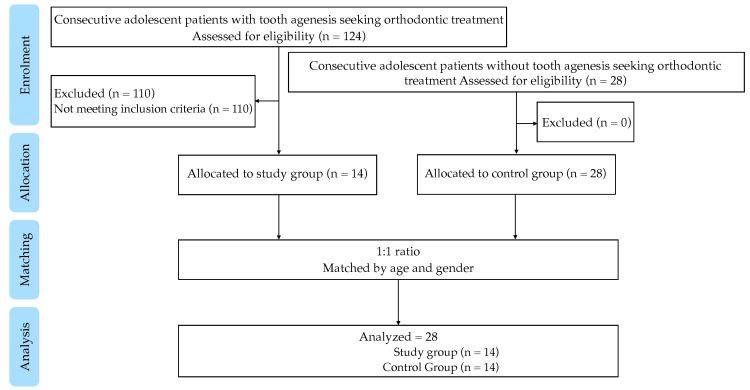
Flowchart of participants.

A flowchart of participants is shown in Figure 3. A total of 34035 sweat pores were counted on 168 fingerprints of 28 study participants. The study group consisted of 14 participants (range 8.4–15.1 years), including nine females (mean age 13.5 ± 3.3 years) and five males (mean age 13.4 ± 4.3 years). The matched control group also consisted of 14 participants (range 7.7–19.3 years), including nine females (mean age 12.4 ± 2.1 years) and five males (mean age 13.4 ± 1.2 years). Baseline characteristics for both groups are shown in Table 1. There was no statistically significant difference between the groups for age, height, weight, and body surface area (BSA). In the study group, the mean number of missing teeth, excluding third molars, was 11.07 ± 4.03.

Intrarater reliability for the primary outcome was excellent, with an ICC of 0.945 (95% CI: 0.860–0.979). In the study group, a statistical analysis using ANOVA indicated no significant differences between individual fingers for the number of pores (p=0.561), the number of pores per cm^2^ adjusted by BSA (p=0.910), the number of measured lines (p=0.807), and the distance (μm) between two consecutive pores on a dermal ridge (p=0.676).

In the control group, the individual fingers differed significantly in the number of pores (ANOVA, p=0.047) and the number of measured lines (ANOVA, p=0.035). A post-hoc Tukey test (assuming equal variances) showed a tendency (p=0.061) for different pore numbers between the right index finger (r2) and the left ring finger (l4). The number of measured lines differed significantly (p=0.036) between the right index finger (r2) and the right ring finger (r4). Since these measurements differed only in the control group, a subgroup analysis was not performed for this study.

The unadjusted number of pores showed a statistically significant difference between the groups. After adjustment for BSA, this difference was no longer present (Table 2). Regarding the distance measurements between consecutive pores on a dermal ridge, the groups differed significantly, not only in the number of lines placed (p=0.013) but also in the distance in pixels (p=0.006) and in the distance calculated in micrometers (p=0.006). The number of lines was lower in the study group, which is related to a larger distance between successive pores. This is confirmed by the distance in pixels and micrometers, which was significantly larger than in the control group.

When differentiating between the right and left hands, there was a trend towards fewer pores and, thus, a greater distance between the pores in the study group. However, statistically significant differences were observed only for the left hand (Table 3).

**Figure 4:**
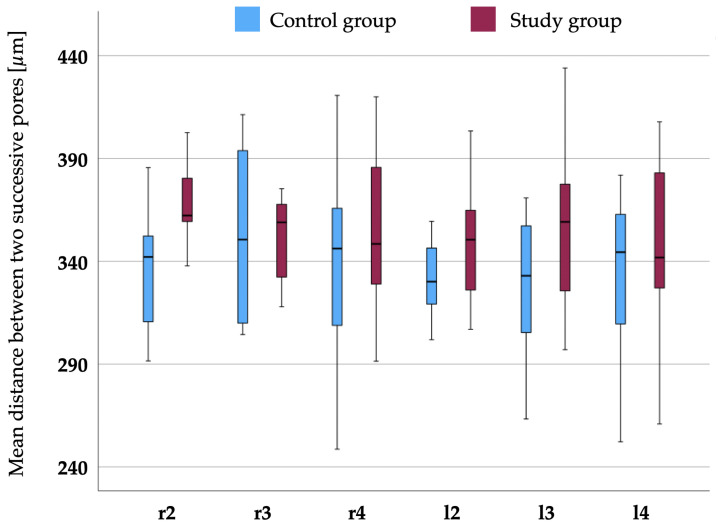
Mean distance between two consecutive pores measured in micrometers on the right (r) and left (l) index (2), middle (3), and ring (4) fingers between the study and control groups. The study group showed larger mean values, indicating fewer pores on a dermal ridge. However, statistically significant larger distances were only found for r2 (p=0.014) and as a trend for l3 (p=0.062).

Figure 4 shows the distance (μm) between two consecutive pores for each finger. Again, the mean values were larger in the study group but significantly larger only for r2 (p=0.014) and as a trend for l3 (p=0.062).

## 4. Discussion

Sweat pore counting has been considered a screening method to identify carriers of X-linked hypohidrotic ectodermal dysplasia [9]. This idea is promising because the embryonic development of the sweat glands is completed at about 22 weeks of gestation and becomes visible on the skin at 32 weeks [19] so that a diagnostic assessment can be made early after birth, long before clinical symptoms such as hypodontia appear. Regarding a possible thermoregulatory disorder, it seems reasonable to assess pore density and function at an early stage, especially if there is a family history of missing teeth.

It is known from numerous studies of ectodermal dysplasia that sweat pore density, and the number of teeth is reduced [20,21,22], but the evidence on the non-syndromic relationship between the number of pores and agenesis of teeth is limited. Confirmation or denial of an association may be helpful in assessing individual risk. Therefore, we compared a group of patients with more than six congenitally missing teeth (excluding third molars) with a matched control group. The number of six teeth was chosen because it has been theorized in the literature that more than six missing teeth may be part of a syndrome or suspected syndrome [23].

We found significantly fewer pores in the study group, but this difference disappeared when the number of pores was adjusted using the BSA. This adjustment was necessary because the static number of pores on the skin surface moves away from each other during growth. However, the adjustment carries the risk of methodological bias because the BSA calculation used is only an approximation of the body surface using geometric bodies. We used Haycock’s calculation [17] because the authors mainly studied children. Another influence to consider is that the BMI of children has increased over the decades since the formula was developed [24,25]. Therefore, the absolute number should not be negated but should be used with caution when interpreting differences. It is common to analyze the absolute number of pores, especially when considering size and gender differences [10,13,26].

Because the distance between sweat pores on one ridge differs between pores on adjacent ridges [13], we measured the distance between successive pores within-ridge only (Figure 2). The distance and the number of placed lines was analyzed. Significantly fewer (p=0.013) lines were placed to connect successive pores on a ridge in the study group. This indicates fewer pores and greater distances, which is confirmed by the measurements in pixels (p=0.006) and micrometres (p=0.006). However, these differences were small and of limited clinical significance. A possible explanation for these differences may be related to the underlying developmental pathways involved in ectodermal structures. Individuals with oligodontia may have alterations in these pathways, resulting in a change in the arrangement of sweat gland structures and the resulting pore density, including the distance between adjacent pores. This increased distance between adjacent sweat pores may suggest a potential compensatory mechanism, as the fixed number of pores may be distributed over a larger area of the dermal ridges, indicating that the observed differences may reflect more general alterations in ectodermal development. Further studies are required to understand the implications for individuals with congenital tooth agenesis.

Due to the fact that pore density varies in different body regions and also from individual to individual [19], normative values are difficult to find in the literature. However, the highest density was found on the fingertips, with 532 pores per cm^2^ [19], making an analysis of this region highly relevant.

Our results in the control group are confirmed by Peters and coworkers [13], who measured a mean pore-to-pore within-ridge distance of 340.19±35.50 μm in 15 healthy participants. Scobbie and Sofaer [6] used an age-based formula and calculated larger distances in the range of 368.64–371.04 μm. It must be noted that almost half of the study population was between 30 and 65 years of age, so the comparison with our data is limited. Sidhu et al. [27] compared a study group affected by ectodermal dysplasia with an unaffected control group (n = 10, mean age 35.8 years), both from the same families. They observed a large variation in pore distances (213.86–421.05 μm) in the control group, which can be explained by family affiliation and cannot be used for comparison with our results.

In forensic science, fingerprint ridges are divided into different levels of detail, with pore distribution, frequency, and pore spacing assigned to level 3 [26,28]. A recent study showed that level 3 features have high reproducibility, and in particular, the frequency distribution of the distance between adjacent sweat pores has a higher identification rate than pore density, even for fingerprint fragments [26]. This is in line with our own results, which show that pore distance has a better discriminative value between the study and control groups than pore density.

The differences observed in the distance between adjacent pores suggest that sweat pore analysis may serve as an effective screening tool for the early identification of individuals at risk for oligodontia, facilitating timely intervention. Incorporating this information into clinical practice could help to personalize treatment strategies and encourage interdisciplinary collaboration between clinicians involved in the management of oligodontia/ectodermal dysplasia.

A strength of this study is the controlled trial design that reduces the influence of potential confounders by employing a well-matched control group. Another strength is the use of scanning electron microscopy, which provides high precision and reliable data. The robustness of findings is further increased by the adjustments for the body surface area (BSA).

However, the study has limitations that need to be considered when interpreting the results. The sample size of 14 participants per group is relatively small, which may affect the generalizability of the findings. The inclusion of participants across a wider age range introduces additional variability that might impact sweat pore density. Geographic and ethnic limitations might also restrict the applicability of the findings to a broader population, as participants were enrolled at one single University Hospital Department in Germany. One potential source of error in our measurement method involves the manual tracing and the precision of the image analysis software used to measure the distances between adjacent sweat pores. Variability in the localization of adjacent pores could lead to measurement inaccuracies. To minimize these errors, we implemented a standardised approach in the image analysis process and ensured that measurements were taken consistently across all samples. Moreover, factors such as the resolution of the scanning electron microscope images and the quality of the fingertip impressions can introduce inconsistencies that may affect the clarity of individual pores. In addition, the use of a specific BSA formula, which may not perfectly represent all body types, could also affect the results. Despite our efforts to minimize potential confounding by using a matched design and consideration of demographic factors such as height, weight, and body surface area, we recognize that other confounding variables remain uncontrolled. Specifically, factors such as ethnicity, environmental influences, and various behavioral characteristics may affect sweat pore characteristics and potentially influence the observed relationship between sweat pore density and tooth agenesis. Future studies with larger sample sizes are necessary to assess possible relations between sweat pore density and tooth agenesis and to improve the external validity of the results.

## 5. Conclusions

Patients with ≥6 congenitally missing permanent teeth differ from subjects without tooth agenesis in the distance between two adjacent sweat pores on a dermal ridge. However, these differences were small and of limited clinical significance. Increased pore distance appears to be a better predictor of oligodontia/ectodermal dysplasia than pore number.

## Figures and Tables

**Table 1:** Baseline characteristics of the control and study groups regarding age [years], height [cm], weight [kg], body surface area (BSA) in [m^2^], number of missing teeth including third molars (mt + M3), and number of missing teeth excluding third molars (mt). Mean values (M), standard deviations (SD), 95% confidence intervals (CI), and *p*-values.

	Control Group			Study Group			
	M	SD	95% CI	M	SD	95% CI	*p*
age	12.79	1.82	11.74–13.84	13.47	3.51	11.44–15.50	0.734
height	160.57	11.57	153.89–167.25	158.07	13.79	150.11–166.03	0.306
weight	52.41	14.77	43.89–60.94	47.68	14.16	39.50–55.85	0.306
BSA	1.52	0.27	1.36–1.67	1.43	0.27	1.28–1.59	0.285
mt + M3	0	0	0	14.86	4.24	12.41–17.31	-
mt	0	0	0	11.07	4.03	8.75–13.40	-

**Table 2:** Number of counted pores, number of pores per cm^2^ adjusted by BSA (pores/BSA), number of lines (lines), and distance between two consecutive pores on a dermal ridge measured in pixels (dist px) and micrometers (dist μm). Mean values (M), standard deviations (SD), 95% confidence intervals (CI), and *p*-values.

	Control Group			Study Group			
	M	SD	95% CI	M	SD	95% CI	*p*
pores	209.71	34.92	202.14–217.29	195.46	31.13	188.71–202.22	0.029
pores/BSA	579.59	197.31	536.77–622.41	565.60	153.51	532.28–598.91	0.583
lines	171.35	37.77	163.15–179.54	150.95	44.51	141.29–160.61	0.013
dist px	61.10	7.01	59.58–62.62	63.72	5.82	62.45–64.98	0.006
dist μm	340.31	39.04	331.83–348.78	354.89	32.41	347.86–361.93	0.006

**Table 3:** Right and left hand differentiation for the number of counted pores (pores), number of pores per cm^2^ adjusted by BSA (pores/BSA), number of lines (lines), and distance between two consecutive pores on a dermal ridge measured in pixels (dist px) and micrometers (dist μm). Mean values (M), standard deviations (SD), 95% confidence intervals (CI), and *p*-values.

	Control Group			Study Group			
right hand	M	SD	95% CI	M	SD	95% CI	*p*
pores	205.45	36.16	194.18–216.72	194.07	32.61	183.91–204.24	0.225
pores/BSA	569.00	199.19	506.93–631.07	562.19	160.83	512.07–612.30	0.700
lines	167.62	44.04	153.89–181.34	149.26	42.30	136.08–162.44	0.080
dist px	62.26	7.42	59.95–64.58	64.26	4.92	62.72–65.79	0.088
dist μm	346.80	41.33	333.92–359.68	357.90	27.43	349.35–366.44	0.088
left hand							
pores	213.98	33.53	203.53–224.43	196.86	29.90	187.54–206.17	0.061
pores/BSA	590.17	197.25	528.70–651.64	569.01	147.70	522.98–615.04	0.648
lines	175.07	30.32	165.62–184.52	152.64	47.07	137.98–167.31	0.099
dist px	59.93	6.45	57.92–61.94	63.18	6.61	61.12–65.24	0.041
dist μm	333.81	35.92	322.62–345.00	351.89	36.81	340.42–363.36	0.041

## Data Availability

The data presented in this study are available on reasonable request from the corresponding author due to ethical reasons.
